# The influence of ginger (Zingiber officinale) on human sperm quality and DNA fragmentation: A double-blind randomized clinical trial

**Published:** 2016-08

**Authors:** Jalil Hosseini, Azar Mardi Mamaghani, Hani Hosseinifar, Mohammad Ali Sadighi Gilani, Farid Dadkhah, Mahdi Sepidarkish

**Affiliations:** 1 *Infertility and Reproductive Health Research Center, Shahid Beheshti University of Medical Sciences, Tehran, Iran.*; 2 *Department of Andrology, Reproductive Biomedicine Research Center, Royan Institute for Reproductive Biomedicine, ACECR, Tehran, Iran.*; 3 *Department of Urology, Shariati Hospital, Tehran University of Medical Sciences, Tehran, Iran.*; 4 *Department of Epidemiology and Reproductive Health, Reproductive Epidemiology Research Center, Royan Institute for Reproductive Biomedicine, ACECR, Tehran, Iran. *; 5 *Department of Epidemiology and Biostatistics, School of Public Health, Tehran University of Medical Sciences, Tehran, Iran.*

**Keywords:** *DNA fragmentation*, *Ginger*, *Semen analysis*, *Infertility*, *Male*

## Abstract

**Background:**

Although the effectiveness of ginger as an antioxidant agent has been exploited, little human research has been conducted on its activity on male reproductive functions.

**Objective:**

This study was designed to investigate the effects of ginger (Zingiber officinale) on sperm DNA fragmentation (SDF) in infertile men.

**Materials and Methods:**

This randomized double-blind, placebo-controlled trial with a 1:1 allocation was performed on 100 infertility treatment candidates who were admitted to Royan Institute for Reproductive Biomedicine, Tehran, Iran. Patients were randomly assigned to receive one of two treatments: ginger and placebo. Patients were given a 3-month oral treatment (members received capsules containing 250 mg of ginger powder twice a day in ginger and a placebo in other group). Before and after treatment, standardized semen samples were obtained to determine sperm concentration, motility, and SDF according to World Health Organization.

**Results:**

There was no significant difference between two groups regarding SDF at baseline (53.48. 95%CI: 37.95-69.02) in cases and (56.75, 95%CI: 40.01-73.5) in controls. The average positive percentage of SDF in patients receiving ginger (17.77, 95%CI: 6.16-29.39) was lower compared with placebo (40.54, 95%CI: 23.94-57.13) after three month of treatment (p=0.02). In multivariate analysis, SDF was significantly lower in patients receiving ginger compared with placebo (mean difference: 3.21, 95%CI: 0.78-5.63, p=0.009). There were no significant differences between two groups regarding to semen parameters.

**Conclusion:**

The present study has demonstrated that ginger in a controlled study of efficacy was effective in decreasing SDF in infertile men.

## Introduction

The genetic integrity of the spermatozoal is prerequisite for normal embryo development and transmission of parental genetic information to the offspring (1). Several studies reported a significant influence of sperm DNA fragmentation on fertilization and pregnancy in animal models (2-5). An increased level of DNA fragmentation may represent a cause of male infertility and lower natural conception, intrauterine insemination (IUI), and IVF outcome rates (6, 7). If the level of DNA fragmentation reduces to below 30%, a couple’s chance of delivering a baby, through IUI treatment, will rises from 1.5% to just 20%(3). Some studies indicate that the treatment with natural antioxidants can significantly reduce the DNA fragmentation levels, improve sperm quality, and increase reproductive efficiency of men (8-10).

The ginger rhizome (Zingiber officinale L., Family Zingiberaceae) that commonly used as a spice contains several biologically active compounds such as gingerol, shogaols, gingerdiol and gingerdione. It is also medically used for its immunomodulatory, anti-tumorigenic, anti-inflammatory, antiapoptotic and antioxidant properties (11). In addition, it was found that Zingiber officinale is associated with a beneficial effect on male reproductive functions in rats, which confirmed by other studies on the increased sperm counts, motility, testosterone, and decreased malonhydialdehyde levels (12-16). 

It was also observed that the administration of ginger can significantly increase the testosterone level in plasma and stimulate spermatogenesis (17, 18). Although the effectiveness of ginger as an antioxidant agent has been exploited in animals, little human research has been conducted on its activity on male reproductive functions (19).

This trial was designed to investigate the effects of ginger rhizome on sperm DNA fragmentation (SDF) and sperm parameters.

## Materials and methods

**Design**

This was a randomized double-blind, placebo-controlled trial with a 1:1 allocation that performed on 100 candidates who were admitted to Department of Andrology at Reproductive Biomedicine Research Center, Royan Institute for Reproductive Biomedicine, Tehran, Iran for infertility treatment between May 2013 and November 2014. All patients had given written informed consent before any study-related tests were done. 

The study was performed in accordance with the Declaration of Helsinki and was approved by the ethical committee of our institute.

**Participants**

Patients were eligible for enrolment of the study if they were aged more than 45 years old, had idiopathic infertility for more than 2 years, presence of fragmented DNA more than or equal to 15% of ejaculated spermatozoa, absence of leukocytospermia, alcohol or drug addiction, and occupational chemical exposure, not using warfarin or other anticoagulant, not using ginger or other herbal medicines and absence of systemic diseases. Exclusion criteria included: a history of post pubertal mumps, a history of epididymal surgery, a history of radiation therapy or chemotherapy, acute epididymitis, unilateral or bilateral subclinical or recurrent varicoceles and an abnormal hormonal profile. Patients were requested to follow a standard diet to avoid effects attributable to ginger intake in food.

**Randomization**

Random number sequence was prepared by independent person using random block sizes of 6. Patients were randomly assigned in two groups, using an interactive voice response system (by phone), based on a computer-generated list of treatment numbers. 

**Interventions**

The study medications were prepared in capsules of identical size and appearance and were packaged by the institute pharmacy according to a randomization list. Patients in the treatment group were given a 3-month oral treatment (capsules containing 250 mg of ginger powder twice a day). Members of the other group received a placebo during the same period. Presence of SDF was assessed in both groups before and after the treatment period. Patients, outcome assessors and statistical analyzer were not unaware of allocation to the treatment or control arm of the study.

**Outcomes**

SDF was considered as a primary outcome and assessed with terminal deoxynucleotidyl transferase-mediated fluorescein-dUTP nick end labeling (TUNEL). The secondary outcomes measured were semen parameters such sperm count, sperm concentration and progressive motility. Semen samples were collected by masturbation into sterile containers after 48-72 hr of sexual abstinence. These samples were delivered at the fertility laboratory within 1 hr after production. World Health Organization (WHO) (1999) consideration was used for clinical semen analysis (21).

**TUNEL assay**

Fragmented DNA in spermatozoa was visualized by TUNNEL assay using a Cell Death Detection kit (Roche, Mannheim, Germany) according to the manufacturer's instruction. Briefly, remaining ejaculated sperm samples were washed from seminal plasma by low-speed centrifugation (600× g; 5 minutes), smeared on microscope slides, air-dried, fixed with 4% paraformaldehyde in phosphate-buffered saline for 60 min, and permeabilized with 0.1% TritonX-100 in 0.1% sodium citrate for 10 minutes at 4^o^C. 

The specimens were incubated in TUNEL reaction mixture in the dark at 37^o^C for 1 hr followed by evaluation in a fluorescence microscope. The percentage of green fluorescing sperm (TUNEL positive) was determined. Two slides were used for the negative (omitting the enzyme terminal transferase) and positive (using DNase I, 1mg/ml for 20 min at room temperature) controls (22).

**Statistical analysis**

This study required the enrollment of 45 patients in each group to have at least 80% power to detect effect size of 3 between treatment and placebo groups regard to main outcome (with two-sided test and type 1 error of 5%). To allow for a 10% drop-out rate, the total number of patients increased to 100. All analyses were performed on an intent-to-treat basis. Summaries of continuous and categorical measures were presented as the mean±SD and n(%) respectively. We compared a difference between baseline characteristics of patients after randomization into the 2 groups with a ^2^test for categorized data and with Student’s t test for continuous variables. 

General linear model (GLM) (family= Gaussian, link= identity) was used to evaluate the two study arms for the primary and secondary end points. The model included treatment as main effects and age, BMI, pre sperm count, pre sperm concentration, pre sperm motility and pre SDF as covariates. Testing was performed at a 95% significance level. Results were presented as the mean difference with 95 percent confidence intervals. Statistical tests were two tailed. Data were analyzed using Stata software version 13 (Stata Corp, College Station, Tex, USA).

## Results

**Figure 1:**
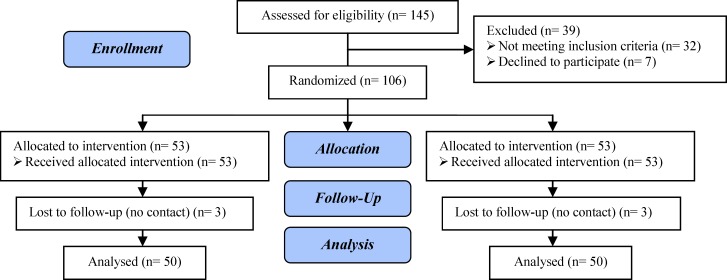
Flowchart showing participants and groups disposition

A total of 145 patients were screened. Only 106 men met the inclusion criteria and were randomly assigned to one of two treatment groups. Three patients dropped out in the treatment arm. Two patients dropped out in the first month and one in the third month. Three subjects in the placebo group discontinued treatment because of lost to follow-up. The study profile is shown in figure 1. 

No significant differences between two groups were observed in patients’ demographics or clinical characteristics (Table I). The percentage of spermatozoa in two groups that was positive for the TUNEL assay was calculated. The average percentage of SDF for two groups is shown in Table II. As can be seen, there was no significant difference between two groups at baseline; 53.48 (37.95-69.02) in cases and 56.75 (40.01-73.5) in controls. The average percentage of SDF in patients receiving ginger (17.77, 95%CI: 6.16-29.39) was lower compared with placebo (40.54, 95%CI: 23.94-57.13) after three month of treatment (p=0.02).

**Figure 2:**
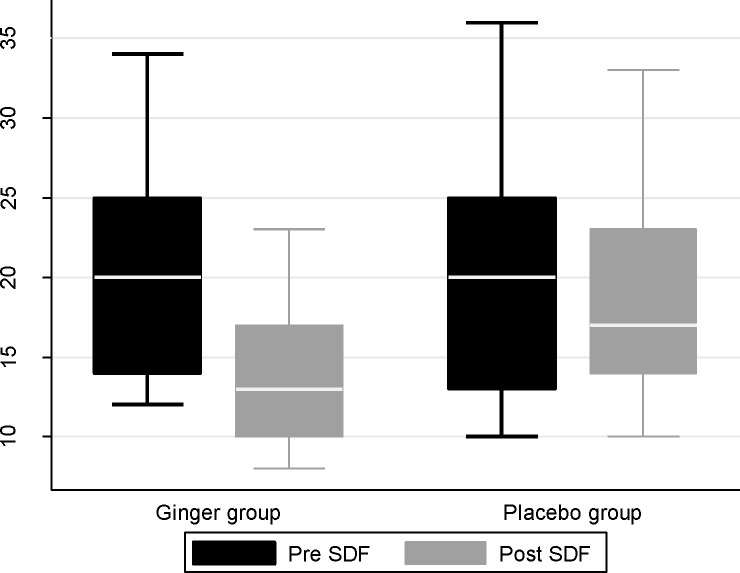
DNA fragmentation between two groups before and after treatment

In bivariate analysis, DNA fragmentation was significantly lower in patients receiving ginger compared with placebo after three month of treatment (mean difference: 4.46, 95%CI: 2.04-6.88, p<0.001) (Figure 2). After adjustment for aforementioned covariates, the difference remained significantly meaningful (mean difference: 3.21, 95%CI: 0.78-5.63, p=0.009). Table III shows the mean differences, corresponding 95% CIs, and p-values for secondary outcomes. As can be seen, there were no significant differences between two groups regarding secondary outcomes. The two groups did not differ with respect to the absolute number of complications and no side effects were observed in two groups.

**Table I:** Baseline demographics and disease characteristics

				**Group 1 (ginger)**	**Group 2 (placebo)**	**p-value**
Age (years)				33.27 ± 5.38	32.05 ± 3.99	0.25
BMI				25.86 ± 3.22	26.47 ± 4.6	0.53
Marriage duration				6.55 ± 3.56	5.73 ± 3.83	0.34
Pre FSH				5.51 ± 3.85	4.25 ± 2.53	0.09
Pre testosterone				4.37 ± 1.92	4.62 ± 1.7	0.52
Pre LH				2.4 ± 1.67	2.84 ± 1.67	0.01
Pre sperm count				131.75 ± 95.58	169.46 ± 127.89	0.82
Pre sperm volume				3.12 ± 1.34	3.52 ± 1.73	0.15
Pre sperm motility				43.09 ± 19.45	41.43 ± 18.19	0.18
Pre sperm liquefaction time				27.23 ± 8.12	25.81 ± 8.12	0.21
Smoking						
			Yes	16 (32)	13 (26)	0.78
			No	34 (68)	37 (74)	
Infertility drug use						
	Yes			41 (82)	44 (88)	0.83
	No			9 (18)	6 (12)	
Testosterone use						
		Yes		4 (8)	5 (10)	0.92
		No		46 (92)	45 (90)	
Vitamin use						
			Yes	10 (20)	14 (28)	0.35
			No	40 (80)	36 (72)	

Values given as mean±SD, or n(%) unless otherwise indicated (n=50).

BMI: Body mass index

LH: Luteinizing hormone

FSH: Follicle-stimulating hormone

**Table II:** The average percentage of sperm DNA fragmentation (SDF) for two groups

	**Group1 (ginger)**	**95% CI**	**Group 2 (placebo)**	**95% CI**	**p-value**
Pre SDF	53.48 ± 0.07	37.95-69.02	56.75 ± 08	40.01-73.5	0.77
Post SDF	17.77 ± 0.05	6.16-29.39	40.54 ± 0.08	23.94-57.13	0.02

Values given as mean±SE with 95% CI (n=50).

SDF: Sperm DNA fragmentation

**Table III:** Secondary outcomes on bivariate and multivariate analysis

	**Crude mean difference (95% CI)**	**Adjusted mean difference (95% CI)** ^*^	**p-value (adjusted model)**
Sperm count	1.21 (-11.08, 13.5)	1.08 (-9.13, 11.3)	0.83
Sperm concentration	0.2 (-0.52, 0.94)	0.11 (-0.46, 0.69)	0.7
Progressive motility	-3.91 (-13.3, 5.47)	-7.79 (-16.13, 0.54)	0.06
Sperm morphology	-1.67 (-3.55, 0.19)	-1.41 (-2.85, 0.02)	0.06

* The model include age, BMI, pre sperm count, pre sperm concentration, pre sperm motility and pre SDF.

## Discussion

The first major finding of our study was the demonstration of a statistically significant negative correlation between the usage of ginger and DNA fragmentation percentage. The study shows that ginger powder in doses of 250 mg twice a day after 3 months is effective in reducing DNA fragmentation as compared with controls. This is the first direct demonstration of protective effect of ginger upon sperm DNA integrity in human although such an effect has previously been suggested with the use of other antioxidant (9, 23, 24).

The primary functions of the testes are to produce sperm (spermatogenesis) and to produce androgens, primarily testosterone (steroidogenesis). Considering normal circumstances, some conditions that can prevent spermatogenesis and decrease sperm quality and quantity include medication, chemotherapy, toxins, polluted air, lack of nutrients and vitamins, which are associated with adverse effects on spermatogenesis and sperm production. These conditions predispose sperm cell susceptible to injury through several pathways and can significantly affect both sperm quality and quantity (2). Apoptosis may be also observed in a natural or normal spermatogenesis. Normal spermatogenesis is adjusted appropriately and the balance between cell proliferation continuously and apoptosis (25).

It has been confirmed that environmental risk factors can lead to apoptosis in sperm and an increase in ROS generation and consequently death of spermatozoa. A disturbance in the pro-oxidant/antioxidant system has been defined as oxidative stress. ROS are reactive molecules ranked as free radicals owing to the presence of one unpaired electron such as a superoxide ion (O-2), nitrogen oxide (NO) and hydroxyl radical (HO-) (5, 26). 

However, ROS naturally exists in the organism, but they are mainly confined to cell compartments and counterbalanced by natural antioxidant molecules, including glutathione, glutathione peroxidase, superoxide dismutase, and vitamin E and vitamin C, behaving as free radical scavengers (27). This oxidative stress-induced sperm damage has been proposed to be an influential contributing factor in more than half of all cases of male infertility (28).

Sperm cell plasma membrane is also different from most of other cell membranes in lipid composition. It contains high amount of polyunsaturated fatty acids (PUFA), especially diPUFA (phospholipids esterified with two PUFA). This unique structure of sperm, resulting in greater sensitivity to the environmental hazards, compared with other cells (29).

ROS is able to reduce axonemal protein phosphorylation and sperm immobilisation, both of which are associated with a decrease in membrane fluidity, through propagating PUFA hydroperoxidation. It can also diffuse into the cells and inhibit the activity of glucose-6-phosphate dehydrogenase (G6PD), which is known a key enzyme in control of the intracellular availability of NADPH-dependent antioxidant enzymes (24).

In chemical analysis of ginger, it was found that it contains over 400 different compounds. Carbohydrates (50-70%), lipids (3-8%), terpenes and phenolic compounds are the major constituents in ginger rhizomes. Terpene components of ginger are zingiberene, β-bisabolene, α-farnesene, β-sesquiphellandrene and α- curcumene, but p gingerol, paradols, and shogaol are considered henolic compounds. There are the gingerols (23-25%) and shogaol (18-25%) in higher quantity compared with others. Z. officinale crude plant material includes amino acids, raw fiber, ash, protein, phytosterols, vitamins (e.g., nicotinic acid and vitamin A) and minerals. It also contains single constituents, such as [6]-gingerol [6]-paradol, phenolic1,3-diketones, zingerone, which are associated with a protective effect against lipid peroxidation in different established models(11, 27).

Identification of ginger anti-oxidative components has been explored in various in-vitro tests and in several antioxidants with a same performance (26). Shogaol, also known as (6)-shogaol, is a pungent component of ginger has exhibited the most towering antioxidant and anti-inflammatory properties in ginger, which can be attributed to the presence of alpha, beta-unsaturated ketone moiety. Zingerone like shogaol is another antioxidant component of ginger that produced when ginger is dried or cooked. In previous studies, there are scavenging effects of zingerone from ginger against intracellular RS (reactive species). Ginger also contains [6]-gingerol, 8-gingerol, 10-gingerol and 12-gingerol, which have similar anti-oxidate properties (27, 31). Animal modeling showed that ginger significantly lowered induced lipid peroxidation and amplified the levels of antioxidant enzymes, quantity and quality of sperm and plus serum glutathione (9, 16, 17, 32).

In a study conducted by Hafez in Greece on the effect of a ginger and cinnamon combination on infertile diabetic rats, a significant increase was observed in sperm parameters and reproductive behavior in terms of sperm parameters including count, motility and viability (15). Another study was conducted by Abo-Ghanema *et al* using the combination of ginger and L-carnitine to treat infertile rats. The authors showed that this combination increased the weight of testicles and seminal vesicles, improved the quality and quantity of semen (33).

In contrast to the major impact on sperm DNA fragmentation, our findings did not show any significant improvement of sperm concentration, motility and morphology after in vivo ginger treatment. Several human studies like aforementioned study also failed to observe an improvement in basic sperm parameters after antioxidant treatment (8, 24).

However, in a study conducted in 2012 by Al-Kadir Mares and Najam at the University of Tikrit, Iraq, a significant increase was observed in sperm count in infertile men after treatment with ginger. In this clinical trial 75 infertile patients were treated by ginger. There was a significant increase in sperm count of infertile men after treatment with ginger as compared with before treatment (19). This inconsistency may be attributed to the design of their study. Their study was performed in before and after design without random allocation. It is possible that their results were affected by baseline value of sperm parameters and regression to the mean.

Previously published data on the effects of antioxidants on sperm concentration, motility and morphology are contradictory. The observed differences between aforementioned studies are likely to be related to the type and dose of antioxidant used, characteristics of the patient group under treatment and the duration of the treatment.

Our results provided evidence for Tesarik *et al* who reported DNA fragmentation levels were decreased and sperm parameters did not show a significant change after antioxidant treatment (vitamin C and E) (3). In their study sixty-four men with unexplained infertility and an elevated (≥15%) percentage of DNA-fragmented spermatozoa in the ejaculate were randomized between an antioxidant treatment (1 g vitamin C and 1 g vitamin E daily for 2 months) group and a placebo group. The percentage of DNA-fragmented spermatozoa was markedly reduced (p<0.001) in the antioxidant treatment group after the treatment (9.1±7.2) as compared with the pretreatment values (22.1±7.7).

In another study that conducted by Josep Gual-Frau, like our study, SDF decreased after antioxidant usage: Twenty infertile patients with grade I varicocele were given multivitamins (1500 mg L-Carnitine, 60 mg vitamin C, 20 mg coenzyme Q10, 10 mg vitamin E, 200 μg vitamin B9, 1 μg vitamin B12, 10 mg zinc, 50 μg selenium) daily for three months. After treatment, patients showed an average relative reduction of 22.1% in sperm DNA fragmentation (p=0.02) and had 31.3% fewer highly degraded sperm cells (p=0.07). Total numbers of sperm cells increased (p=0.04) but other semen parameters were unaffected (24). 

This study has several limitations; the most important one is that the number of participants studied was relatively small. Our sample size calculation was based on the main objective of the study and perhaps one of the reasons for the lack of a significant change in sperm parameters is insufficient power. The three months period of treatment in this study might not have been sufficient for discovering all effects of ginger components and only one dose of ginger was given to the patients. It is suggested that future clinical trials might look into larger, dose-response and longer therapy by ginger extracts. 

In spite of this animal in vivo and in vitro studies have shown that ginger usage leading to reduced sperm DNA fragmentation, therefore we recommend that a larger randomized, placebo controlled trial on the efficacy and safety of this valuable antioxidant should be done. Nevertheless, our findings suggest new path of future infertility research and treatment. 

## Conclusion

The present study has demonstrated that ginger in a controlled study of efficacy was effective in decreasing SDF in infertile men. Although its beneficial effect on fertility remains to be established, this finding opens avenues of future fertility research and treatment, and may affect public health.

## note

***Registration ID in Clinical Trial gov:*** ***NCT02032251***
